# Implication for Cancer Stem Cells in Solid Cancer Chemo-Resistance: Promising Therapeutic Strategies Based on the Use of HDAC Inhibitors

**DOI:** 10.3390/jcm8070912

**Published:** 2019-06-26

**Authors:** Maria Serena Roca, Elena Di Gennaro, Alfredo Budillon

**Affiliations:** ExperimentalPharmacology Unit, Istituto Nazionale Tumori–IRCCS–Fondazione G. Pascale, 80131 Naples, Italy

**Keywords:** cancer stem cells, solid cancer, chemo-resistance, HDAC inhibitors

## Abstract

Resistance to therapy in patients with solid cancers represents a daunting challenge that must be addressed. Indeed, current strategies are still not effective in the majority of patients; which has resulted in the need for novel therapeutic approaches. Cancer stem cells (CSCs), a subset of tumor cells that possess self-renewal and multilineage differentiation potential, are known to be intrinsically resistant to anticancer treatments. In this review, we analyzed the implications for CSCs in drug resistance and described that multiple alterations in morphogenetic pathways (i.e., Hippo, Wnt, JAK/STAT, TGF-β, Notch, Hedgehog pathways) were suggested to be critical for CSC plasticity. By interrogating The Cancer Genome Atlas (TCGA) datasets, we first analyzed the prevalence of morphogenetic pathways alterations in solid tumors with associated outcomes. Then, by highlighting epigenetic relevance in CSC development and maintenance, we selected histone deacetylase inhibitors (HDACi) as potential agents of interest to target this subpopulation based on the pleiotropic effects exerted specifically on altered morphogenetic pathways. In detail, we highlighted the role of HDACi in solid cancers and, specifically, in the CSC subpopulation and we pointed out some mechanisms by which HDACi are able to overcome drug resistance and to modulate stemness. Although, further clinical and preclinical investigations should be conducted to disclose the unclear mechanisms by which HDACi modulate several signaling pathways in different tumors. To date, several lines of evidence support the testing of novel combinatorial therapeutic strategies based on the combination of drugs commonly used in clinical practice and HDACi to improve therapeutic efficacy in solid cancer patients.

## 1. Introduction

Drug resistance is a well-known phenomenon that arises when a disease becomes tolerant to treatment. This concept was described first in bacteria when they became resistant to antibiotics, but since then, this phenomenon has been observed in other diseases, including cancer. Although many types of cancers are initially susceptible to antitumor approaches including chemotherapy and target-therapy and immunotherapy, over time, cancers can develop resistance through several mechanisms, such as DNA mutations or metabolic changes that promote drug inhibition and degradation [1]. Solid tumors are biologically complex structures with strong intratumor heterogeneity that arises among cancer cells within the same tumor as a consequence of genetic changes, environmental differences, and epigenetic and reversible changes in cell features [2]. Two main conceptual frameworks have been elaborated to conceptualize the link between intratumor heterogeneity and therapy resistance [3]. The first and most supported idea is clonal evolution, where a single mutated cell creates a tumor and over time acquires additional mutations, resulting in several subpopulations with evolutionary advantages [4]. An example of this comes from the analysis of circulating tumor DNA in the blood of colorectal cancer (CRC) patients with primary or acquired resistance to epidermal growth factor receptor (EGFR) blockade [5]. Siravegna and colleagues exploited the circulating tumor DNA to genotype colorectal tumors and tracked clonal evolution during treatment with EGFR-specific antibodies, discovering that the percentage of mutated KRAS clones declines in blood when EGFR-specific antibodies are withdrawn. This result suggests that resistant cell populations are highly dynamic and that specific resistant clones arise in specific therapeutic conditions [5].

**Figure 1:**
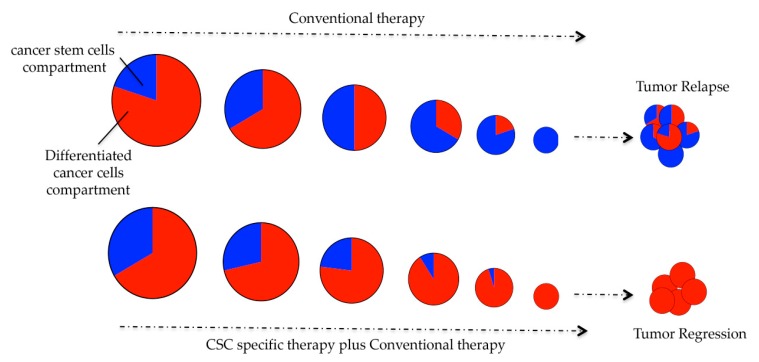
Cancer stem cells (CSCs) model of drug resistance:CSCs are responsible for tumor heterogeneity, drug resistance and tumor relapse. Indeed, they may survive chemotherapy lead to tumor relapse. Only by taking advantages of a CSCs specific targeted therapy, the outcome could result in tumor regression and patients’ complete survival.

The second concept is the cancer stem cell (CSC) model. In the last decade, several lines of evidence have suggested the presence of CSCs within the plethora of heterogeneous cells in solid cancers. The CSC paradigm implies that the tumor is organized into a hierarchy of subpopulations of tumorigenic CSCs and their non-tumorigenic progeny [6]. Among other described CSC features, self-renewal potential and the capability to generate progenitor/daughter cells with various degrees of differentiation make CSCsresponsible for tumor heterogeneity, drug resistance and tumor relapse (Figure 1) [7]. However, although several papers have identified CSCs as being responsible for drug resistance, the evidences are based on the identification and characterization of CSCs made with numerous non-homogeneous experiments that are difficult to compare. Thus, to confirm this theory, different approaches (*in vitro*, *in vivo* and *in silico*) must be implemented. Moreover, understanding the features and the complex signaling mechanisms that underlie the CSC state is a key point in highlighting new possible therapeutic strategies to target CSCs and to overcome resistance (Figure 1).

To date, it is widely recognized that alterations of the “epigenome” are therapeutically relevant as well as DNA mutations. Indeed, in contrast to DNA mutations, “epimutations” must be actively maintained through DNA replication for their dynamic nature; thus, their functional effects are reversible and, consequently, targetable [8,9].

Increasing evidence supports the significance of the epigenetic regulation of CSCs’ features [10]. DNA methylation as well as histone acetylation are two epigenetic modifications that participate in the modulation of expression of many genes, regulating important cellular activities such as proliferation, differentiation and migration. While the role of DNA methylation in CSCs is relatively well established, the role of histone and non-histone protein acetylation is still not completely clear.

It has been suggested that, globally, hypoacetylated chromatin is associated with cancer [11]. In detail, as reviewed by Liu et al. [12], the dysregulation of two classes of enzymes, histone acetyltransferases (HAT) and histone deacetylases (HDAC), implicated in the regulation of acetylation levels is related to carcinogenesis and the regulation of the stemness properties of both normal and cancer cells. Thus, acetylation and deacetylation of histones and non-histone proteins regulate important signaling pathways involved in the maintenance of cancer stem-like cell traits such as self-renewal or differentiation.

In invasive breast cancer patients, Sulaiman et al. recently observed a direct link between HDAC expression and CSCs. By analyzing 887 samples, they found that high Wnt and HDAC activity was associated with estrogen receptor 1 (ESR1) and progesterone receptor (PGR) repression, poor survival and increased relapse. Interestingly, clinically achievable doses of Wnt, HDAC, and ESR1 inhibitors were able to inhibit both bulk and CSC subpopulations, inducing the differentiation of CSCs in non-CSCs without affecting normal mammary epithelial cells (MCF-10A) [13]. The overexpression of individual HDACs has been correlated to predict poor patients’ prognosis independently of tumor type and disease stage and a role of specific HDACs has been also reported in CSCs. The genetic knockdown of individual isoforms such as HDAC1, 2, 3 and 6 have been shown to induce cell cycle arrest and apoptosis in several tumor types including breast, lung, colon cancer and leukemia as review by West et al. [14]. Witt et al. [15] taking advantage of two independent pairs of genetically matched immortalized breast cancer cell lines, derived one from normal human breast precursor epithelial cells with a mixed luminal-myoepithelial phenotype resembling CSC characteristics, and the one from normal human mammary epithelial cells that exhibit a more differentiated myoepithelial phenotype, demonstrating that HDAC1 and HDAC7 play an essential role in the stem-like phenotype, maintaining the CSC population in breast and ovarian cancer models. Another work reported that the levels of HDAC 1, 7 and 8 were overexpressed in pancreatic ductal adenocarcinoma (PDAC) compared to those of adjacent non-tumor tissues and that patients with high levels of HDAC 1, 7 or 8 had significantly worse overall survival compared to those with low expression levels [16]. A specific role of HDAC8 was also demonstrated in breast cancer, where a selective HDAC8 inhibitor suppressed Notch 1 expression [17]. Moreover, An et al. reported that, in triple negative breast cancer, HDAC8 induced cell migration by Hippo–Yap signaling. In detail, HDAC8 suppressed the phosphorylation of YAPSer127 to promote its nuclear localization [18]. 

In contrast, Zimberlin et al. taking advantage of a mouse model in which HDAC1 and HDAC2 were simultaneously deleted in the intestine of adult mice, showed a rapid loss of intestinal homeostasis and a decrease in stem-like features when both HDACs were deleted [19]. This latter observation suggests a rationale for the intestinal side effects often observed during treatment with HDAC inhibitors (HDACi). Similarly, Jamaladdin et al. by conditional knock-down in embryonic stem cellsdemonstrated the essential role of HDAC1 and 2 in cellular proliferation and stem cell self-renewal throughthe regulation of key pluripotent transcription factors [20]. Indeed, severe phenotypes were observed following specific deletion of both HDAC1 and HDAC2 in the heart, brain, smooth muscle and neuronal endocrine cells, suggesting a fundamental role for these HDACs in organ maintenance [21]. However, the hypothesis of positive or negative effect exerted by HDACi on normal stem cells is controversial. In this regard, Yusoff and colleagues published a systematic review about preclinical studies evaluating the organ protection effects of HDACi. They conclude that HDACi reduce mortality in experimental models by conferring multi-organ protection, often following a single treatment administered in some cases post injury, suggesting the design of early phase clinical trials in order to confirm the protective role exerted by these agents [22].

From this perspective, the increased dependency of tumor cells on HDACs and their upregulation during transformation could help identifying the therapeutic window’s width of HDACi. At the same time, we should be aware of the potential toxicity induced by this class of agent. 

Below, we provide an overview of promising therapeutic strategies based on HDACi, to sensitize CSCs to antitumor approaches including chemotherapy and target and immunotherapy. We first outlined the multiple alterations within morphogenetic pathways that show a critical role in CSC plasticity and are potentially targeted by HDACi. Then, considering all intrinsic and extrinsic features of CSCs responsible for chemo-toxicity escape, we discuss the capability of HDACi, alone or in combination, to overcome chemo-resistance. 

## 2. Morphogenetic Pathways Are Dysregulated in Solid Cancers

**Figure 2:**
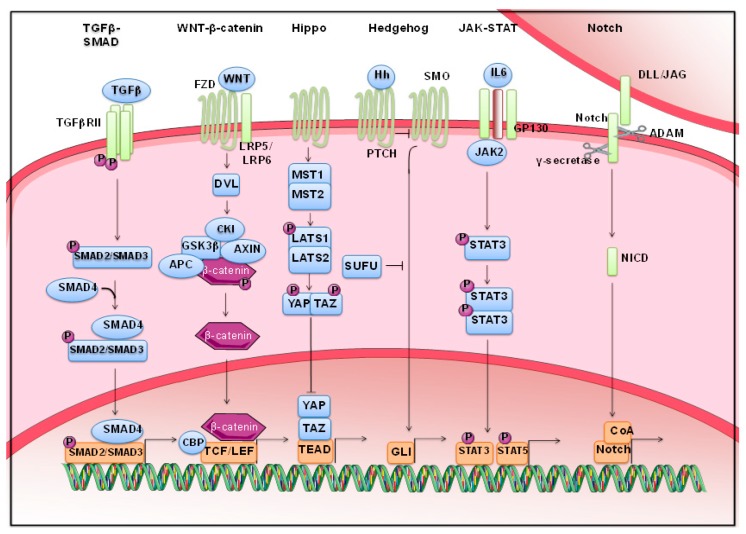
CSCs-activated signaling pathways: Schematic representation of the signaling pathway responsible for induction and maintenance of CSC state. The transcription factors, co-factors, main nodes reported are targetable by drugs. (Adapted from Pattabiraman and Weinberg, Nat Rev Drug Discovery, 2014 [44]).

As extensively demonstrated, stem cell proliferation and cell fate are under the control of several morphogenetic pathways. Among them, we focused on those that have been extensively characterized in cancer: Wnt/β-catenin, Notch, Hedgehog, TGF-β/BMP, JAK-STAT and Hippo pathways (Figure 2). These pathways can be altered in solid cancer and participate in phenomena such as drug resistance. However, these pathways undertake a different role depending on which solid cancer is considered. To identify specific morphogenetic pathways that are more relevant than others in specific solid tumors, we performed a bioinformatics analysis, taking advantage of 16 public solid tumor expression datasets (Table 1 reports The Cancer Genome Atlas (TCGA) Id, the sample size and the patient status for each dataset) by using the online tool R2 Genomics Analysis and Visualization Platform (https://hgserver1.amc.nl/cgi-bin/r2/main.cgi). R2 calculates, for all the genes in the differentiation-related KEGG pathways (718), whether they are differentially expressed considering overall survival, which is the best endpoint to consider to compare multiple cancer types with the goal of identifying common themes that transcend the tissue of origin and may inform precision oncology, as described in a recent publication by Liu et al. [23]. 

We are aware that our pan-cancer systematic analysis is performed across all TCGA tumor types without considering patients clinical features. In details, we did not considered age of diagnosis and grade, when available in TCGA datasets. Thus we anticipated that ouranalysis hassome limitations.

In a subsequent calculation, the overrepresentation of these genes in the individual pathways was determined. A valuewas reported depending on how many related genes were identified withineach pathway as compared with all 718 differentiation-KEGG pathways analyzed. In detail, we tested the significance of the 6 chosen morphogenetic pathways described above. In Table 1, the significance values are reported for the 6 pathways for each solid cancer, and the differences among the 6 pathways analyzed are represented by a color scale. From the resulting list, it is obvious that any specific pathway can be indicated as most relevant in the poor prognosis of patients for all cancer types. Notably, the Hippo pathway had a strong over-representation in patients with a poor prognosis among solid tumors (first in 7/16 tumors), playing a critical role in pancreatic, prostate, stomach adenocarcinomas, lung squamous and cervical cell carcinomas, sarcoma and skin cutaneous melanoma. Similarly, the Wnt/β-catenin pathway seems to play a very important role, representing the most important pathway in 4/16 cases, such as mixed colon adenocarcinoma, liver hepatocellular, head and neck and bladder urothelial carcinomas and third most-important pathway in 8/16 cases (Table 1). Jak/ STAT resulted as key pathway in rectum adenocarcinoma and glioblastoma, while TGFβ in lung adenocarcinoma, Notch in kidney renal clear cell carcinoma and finally Hedgehog in breast invasive cancers.

### 2.1. Wnt Signaling

Wnt signaling is one of the main pathways regulating development and stemness, and it has also been closely related to cancer. The canonical Wnt signaling pathway, which through β−catenin modulates the expression of specific target genes, is an important regulator of stem cells and CSCs and is aberrantly activated during the development of various human cancers as reviewed by Fodde et al. [24]. Gain-of-function mutations of the *CTNNB1* gene (encoding β−catenin) and loss-of function mutations of *APC* and *AXIN* genes were identified as the main mechanisms associated with Wnt signaling dysfunction in cancers [25]. The role of this pathway in carcinogenesis has been most prominently described for colorectal cancer (CRC). In the gut, the Wnt pathway is essential for sustaining cell proliferation within the crypt base and shows a gradient in expression along the crypt axis. Manipulation of Wnt signaling can drastically alter crypt integrity, while the stimulation of Wnt activity by, for instance, R-spondin leads to crypt proliferation [6], and its inhibition leads to the loss of crypt formation and progenitor offspring [7]. Moreover, the presence of Wnt in conjunction with other morphogenetic pathways is critical for maintaining normal and cancer cycling stem cells and Paneth cells, as reviewed by Reya and Clevers [26].

### 2.2. Hippo Pathway

In mammals, cell–cell junctions and apicobasal polarity are involved in upstream activation of the Hippo cascade. The core of the Hippo pathway is a kinase cascade: the nuclear result is that Lats1/2, nuclear dbf2-related family kinases, phosphorylate two major downstream effectors, Yes-associated protein (YAP) and transcriptional coactivator with PDZ-binding motif (TAZ), resulting in their ubiquitination and proteolysis [27]. Nguyen et al. showed that YAP1 induced transcriptional changes and resulted in age-related prostate tumors in mice [28]. Similarly, Zhang et al. showed a significant upregulation and hyperactivation of YAP in castration-resistant prostate tumors compared to their levels in hormone-responsive prostate tumors. They claimed that the enhanced expression of YAP was able to transform immortalized prostate epithelial cells and promote migration and invasion in both immortalized and cancerous prostate cells [29].

### 2.3. Stats

Stats are a family of transcription factors with additional functions in the cytoplasm, the mitochondria and the nucleus. They participate in chromatin conformation and epigenetic marking in the nucleus. In addition, they affect oxidative metabolism in the mitochondria and in the cytoplasm, and they interact with microtubule components to regulate cellular motility [30]. However, in our bioinformatics analysis, the JAK/STAT pathway was highly represented in glioblastoma and rectum adenocarcinoma (Table 1). Signal transducer and activator of transcription 3 (STAT3) is aberrantly activated in glioblastoma and has been identified as a relevant therapeutic target in this disease and many other human cancers [31]. Moreover, Gangulyand colleagues demonstrated high levels of STAT 3-phosphorylation in Tyrosine 705 and Serine 727 in glioma-initiating cells compared to their differentiating counterparts, suggesting a clear involvement of STAT pathway activation in gliomagenesis [32]. In addition, a strong link between IL-6-STAT3 signaling and O6-methylguanine DNA methyl transferase expression and methylation and to temozolomide sensitivity in glioblastoma was found, suggesting a possible combination therapeutic approach based on IL-6/STAT3 inhibitors [33,34]. Nevertheless, STAT3 is over-activated in many breast cancers, while STAT5 promotes both survival and differentiation of mammary epithelium. Moreover, it is known that, in the context of breast cancer, STAT3 activity can be modulated through STAT5 activity, and their combined functions can have an impact on breast cancer progression [35].

### 2.4. TGF-β Signaling

TGF-β signaling controls the cell cycle, differentiation and the microenvironment in epithelial cells through both the SMAD protein family and non-SMAD signaling pathways. In colon cancer, Yusra et al. showed that the expression levels of TGF-β receptor genes, TGFBR1 and TGFBR3, were higher in CD133 positive (+) colon CSCs, suggesting that CD133+ colon CSCs have increased sensitivity to TGF-β [36]. They showed high expression of the *TGFBR1* gene in the tumor budding structures derived from the mouse xenograft with TGF-β transfected cells. Importantly, Yu and colleagues showed that *TGFBR2* was responsible for triggering the TGF-β signaling pathway through recruitment and phosphorylation of Type I receptor, which has been shown to act as a tumor suppressor by assisting in the regulation of stemness through downregulated signaling effects of the Wnt/β-catenin signaling pathway [37].

### 2.5. Notch Pathway

Regarding the Notch pathway, the literature provides many data about its fundamental role in development and maintenance of the hematopoietic system. Less is available on its role in solid cancers. In hematopoiesis, Notch plays a central role in cell fate proliferation and differentiation of stem cells. There is also evidence demonstrating that the Notch pathway can leads hematopoietic stem cells and common lymphoid precursors to undergo T- or B-cell differentiation [38]. Moreover, Notch alteration has been associated with tumorigenesis, since it can be considered, depending on cell model, as an oncogene or a tumor suppressor [39].

### 2.6. Hedgehog Pathway

Finally, the Hedgehog (Hh) pathway is mainly involved in tissue repair, embryonic development, and epithelial-to-mesenchymal transition in cells. In the signaling cascade, Hh ligands, such as Sonic Hedgehog (Shh), Indian Hedgehog (Ihh), and Desert Hedgehog (Dhh), undergo cleavage and produce a signaling protein with dual lipid modifications. Subsequently, the signaling cascade initiated by smoothened (SMO) leads to activation and nuclear localization of GLI transcription factors, which drive the expression of Hh target genes, mostly involved in proliferation, survival, and angiogenesis [40]. This signaling transduction pathway has been shown to be required for self-renewal and proliferation of cerebellar, retinal, and pancreatic CSCs [41]. Recently, Duan et al. have shown that Hh signaling is upregulated in breast cancer cells through nuclear factor-κB (NF-κB) activation and promoter hypomethylation. In addition, overexpression of Shh enhances the self-renewal capacity and migration ability of breast cancer cells and is a poor prognostic indicator in breast cancer [42].

Because of a clear deregulation is described in multiple cancer types, various clinical approaches have been aimed to target the aforementioned pathways. Although several types of inhibitors of the Wnt-pathway are under development as single-agent anticancer therapies, they have not led to exciting results thus far. Indeed, even if PRI-724 (CBP/β-catenin antagonist), LGK-974 (Porcupine inhibitor), vantictumab (anti-Frizzled-1/2/5/7/8 antibody), OMP-54F28 (Frizzled-8–Fc decoy fusion protein) and TSA101 (a radiolabeled anti-Frizzled-10 antibody) are all currently in early clinical development, the results obtained in three concluded studies in monotherapy with PRI-724 (NCT01302405), vantictumab (NCT01345201) and OMP-54F28 (NCT01608867) are not very promising [42]. 

In solid tumors, several clinical trials are ongoing to test the efficacy of two major classes of Notch inhibitors such as γ-secretase inhibitors, which obstruct Notch receptor cleavage, and monoclonal antibodies that interfere with the Notch ligand-receptor interaction, as reviewed by Venkatesh et al. [38]. Thus far, the best results have been obtained in a clinical trial that ended in 2012, where a γ-secretase inhibitor (MK-0752) was used in adult patients with advanced solid tumors, mostly gliomas. Only one objective complete response and stable disease longer than 4 months in 10 patients were observed among 113 patients enrolled [43].

Limited results have been obtained with vismodegib, an Hh pathway inhibitor, which was approved by the European Medicines Agency (EMA) in 2013 for the treatment of metastatic basal cell carcinoma (BCC) or locally advanced BCC in patients who are not candidates for surgery or radiotherapy. Indeed, the gain in overall survival in these patients compared to standard therapy, is only 0.8 years [42].

Currently, several other attempts are ongoing, targeting CSCs to deplete the tumor; however, to date, no specific CSC targeted therapy is yet available. In contrast, it may be more effective to use an approach based on pushing CSCs to differentiate and on sensitizing them to chemotherapy by modulating their epigenetic profile. Indeed, by highlighting the significance of the epigenetic profile in CSC maintenance, in the next sections we reviewed the role of HDACi to target CSCs, both directly and through the modulation of the morphogenetic pathways described above. Actually, by demonstrating that morphogenetic pathway alterations correlating with poor prognosis often coexist in a solid cancer type, we anticipated the limited efficacy of targeted agents inhibiting a single pathway as reported above. This could shed a new light on therapeutic agents with pleiotropic effect such as HDACi. Therefore, we next discussed how HDACi could play a role in overcoming resistance to some of the most commonly used chemotherapeutics and their ability to enhance specific cancer therapeutic agents by targeting CSCs.

## 3. Histone Deacetylase (HDAC) Inhibitors Target Cancer Stem Cells (CSCs) and Morphogenetic Pathways

**Figure 3:**
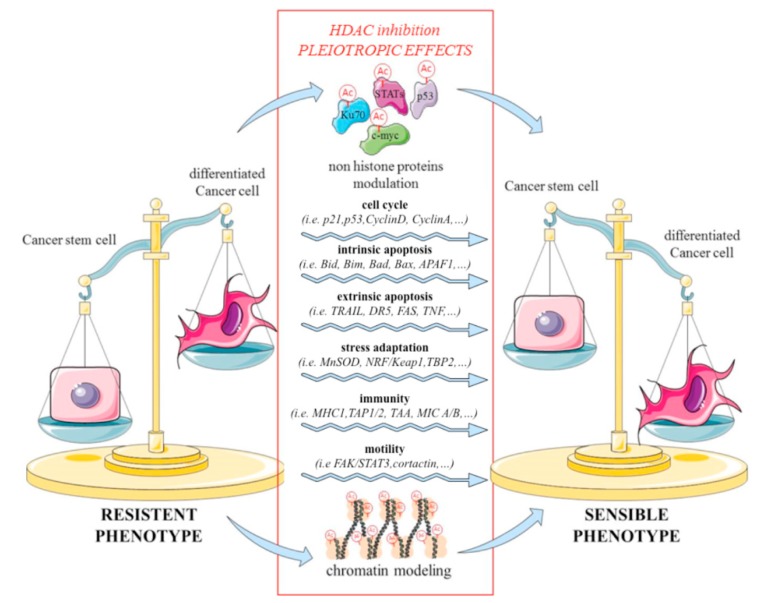
Histone deacetylase inhibitors (HDACi) pleiotropic effects toward CSCs: HDAC are described to modulate several pathways involved in cell cycle, metabolism, stress adaptation, intrinsic and extrinsic apoptosis, motility and immunity through chromatin modeling and non histone proteins modulation. The plasticity of epigenetic regulation makes HDAC inhibition a good strategy to target CSCs chemo-resistant subpopulation in solid cancer pushing the CSCs to differentiate and so gain a chemo-sensible phenotype.

It is well known that the morphogenetic pathways described above are frequently dysregulated in cancer by epigenetic mechanisms as reviewed by Munozet al. [45]. Since trichostatin A (TSA), a natural product isolated from *Streptomyces hygroscopicus*, was described as an HDACi [46], several agents of this class of epigenetic drugs have been synthetized and tested as anticancer agents, both in preclinical and clinical models. HDACi are truly pleiotropic agents, which act through a wide variety of disparate and mutually interactive mechanisms. Indeed, by influencing the chromatin structure, HDACi regulate gene expression. Moreover, by acting on non-histone proteins deacetylation, HDACi can also modulate cellular functions independent of gene expression. In this way, HDACi regulate different altered pathways in cancer, such as apoptosis, DNA repair, growth arrest, terminal differentiation, senescence, apoptosis, anti-angiogenesis, anti-metastasis and immune responses, all of which represent hallmarks of cancer and critical characteristics of CSCs [11]. Finally, HDACi have a strong effect on CSCs by pushing them from a stem-like and resistant phenotype to a more differentiated and sensible phenotype (Figure 3). 

In recent years, we demonstrated that HDACi, such as vorinostat and valproic acid (VPA), were able to induce selective death of the CSC subpopulation in non-small-cell lung cancer (NSCLC) and CRC models, as evaluated by colony and sphere formation assays [47,48]. 

Similar results were obtained by Salvador et al. who demonstrated that the HDACi abexinostat induced cytotoxicity in 16 breast cancer cell lines (BCLs), as evaluated by Aldefluor and tumorsphere formation assays. They found that abexinostat induced CSC differentiation, identifying the long non-coding RNA Xist (Xinactive specific transcript) as a biomarker that can predict the BCL response to HDACi [49]. In line with these findings, Cai et al. demonstrated that TSA or vorinostat, downregulating HDACs 1, 7 and 8, repressed epithelial to mesenchymal transition (EMT) in PDAC and targeted well-known CSC phenotypes, including resistance to therapy and metastasis. Notably, they also suggested that HDACi such as mocetinostat, which specifically inhibits isoforms 1, 2, 3 and 11 HDACs, were not good candidates for PDAC treatment [16]. Moreover, we obtained preliminary data showing that HDACi sensitized cancer cells to chemotherapeutics by pushing them in a differentiation state in colorectal and prostate cancer cell models by modulating morphogenetic pathways [50].

In addition to the preclinical studies that focused on HDACi and their functional effects on modulating CSC subpopulations, several attempts are aiming to explain the molecular mechanisms underlying HDACi effects of HDACi on them. We most likely believe that HDACi strongly modulate the CSC phenotype through their ability to modulate one or more morphogenetic pathway main components, as described above.

Similarly, several data support the close connection between HDAC6 and multiple components of the Wnt, Hippo, TGFβ and Hh pathways. Concerning the Wnt pathway, two papers showed that HDAC6 inhibition led to a decrease in β-catenin nuclear localization, resulting in a strong inhibition of cell proliferation [51,52]. In breast cancer, the involvement of HDAC6 in the Hippo pathway-regulating network was demonstrated by downregulation of YAP protein levels [53] and by a strong acetylation and destabilization of the tumor suppressor MST1 [54]. Some evidence suggests that HDAC6 plays a role in theTGFβ−SMAD activation pathway, and thus its inhibition establishes a decrease in EMT induction [55,56]. Finally, most recently, it has been shown that HDAC6 inhibition leads to inactivation of Gli1, resulting in glioma cancer cell radio-sensitization [57]. In the same manner, HDAC1 has been shownto be essential for SMAD2-3 and Gli1 transcriptional activation, consequently also quenching related pathways [58,59]. As expected, the molecular mechanism becomes complicated when we consider the effect of HDACi, which target more than one HDAC isoform and, simultaneously, more than one morphogenetic pathway.

For example, valproic acid (VPA), a class I- II HDAC inhibitor, is clearly able to inhibit TGF-β, Yap and Notch signaling by dual suppression of SMAD4 and SMAD3 [60], by depletion of nuclear YAP [61] and by downregulation of the transcription factor Notch1 and its target gene HES-1 [62]. Moreover, it has been reported that the pan-HDAC inhibitor trichostatin A (TSA) and the selective HDAC inhibitor entinostat (isoforms 1, 2 and 3) are able to reduce Wnt pathway activation by induction of DKK1, a negative regulator of β−catenin [63] and by inhibition of HDAC1-2 [19].

Finally, as VPA and TSA, vorinostat modulates SMAD4 localization and Nocth3 expression [64,65], but Fan and colleagues also showed that a small molecule SMO-HDAC antagonist was able to retain inhibitory activity for Gli transcription induced in SMO-dependent and SMO-independent ways [66]. Furthermore, TSA and domatinostat, two specific class I and II HDAC inhibitors, switched off Gli signaling by downregulating a transcriptional factor of FoxM1 [67] in the first case and by inhibition of HDAC1/2/3 in the second case [68]. However, it should be noted that some papers have reported that VPA treatment is responsible for Wnt pathway activation by β−catenin nuclear stabilization [13,69].

However, the exact function and interactions governing HDACi activity remain elusive. Thus, further investigationsare necessary to understand the mechanism by whichHDACi target CSCs. Only a clear view of their actions will lead to the identification of a biologically efficacious dose. Thus, it should always be considered that the effects of HDACi depend not only on the cancer type but also on the context, dosing and schedule of treatment. For example, the association of entinostat with the aromatase inhibitor exemestane has been designated as a breakthrough therapy for the treatment of recurrent/metastatic estrogen receptor-positive breast cancer based on the results of a phase II randomized trial [70]. In contrast, the clinical activity of mocetinostat, tested in phase I/II in association with gemcitabine in patients with solid tumors including pancreatic cancer, has been considered insufficient to merit further testing in this setting [71], as well as due to the significant toxicities in the phase II cohort [71]. 

## 4. CSC Chemo-Toxicity Escape Mechanisms

Cancer drug-resistance can be due to intrinsic or acquired factors. The intratumor heterogeneity of tumor cells and the tumor microenvironment and the presence of cancer stem cells are all intrinsic characteristics involved in cancer drug resistance. But latter, has also been observed in most drug-sensitive tumor types for most classes of drugs as an acquired mechanism. 

**Figure 4:**
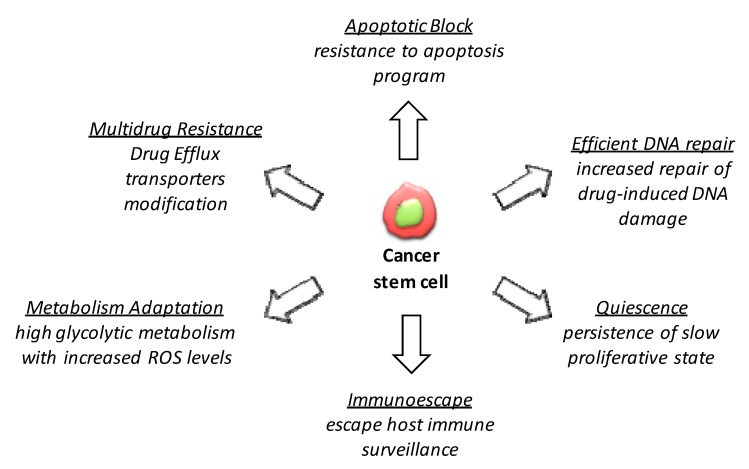
CSCs model of drug resistance: CSCs features mostly responsible for drug resistance.

Several mechanisms of cancer drug resistance are well described in both tumor cells and CSCs, and the most important and targetable ones, such as multidrug resistance [72], resistance to apoptosis program, increased repair of drug-induced DNA damage and quiescence phenotype induction [73], are presented schematically in Figure 4. In addition to the common and well-known mechanisms by which normal stem cells and CSCs overcome drug toxicity, some others have recently been described, such as metabolism adaptation or activation of the immune escape program, while others remain to be uncovered.

The CSC-resistant phenotype is likely to be the result of a complex but specific mixture of molecular circuitries, and it is the complex nature of this phenomenon that explains the difficulties encountered in trying to overcome drug resistance by targeting this specific cell population. 

### 4.1. Multidrug Resistance

Over 40 years ago, multidrug resistance, the high expression of drug efflux pumps, such as ATP-binding cassette (ABC) transporter family proteins, was the first described innate resistance mechanism. Overexpression of ABCB1 confers cancer cell resistance to multiple drugs (i.e., DNA-toxic antitumor agents, reactive oxygen species –ROS- inducers), including colchicine, doxorubicin, etoposide, vinblastine and paclitaxel [74]. The critical role of ABC transporters in CSC drug resistance was reported by Chau et al. in c-Kit+ ovarian CSCs. The authors demonstrated that Wnt/β-catenin regulates ABCG2 expression and resistance to cisplatin/paclitaxel. LEF/TCF binding sites are within the *ABCG2* gene promoter, indicating that ABCG2 is a transcriptional target of β-catenin [75].

Moreover, in a prospective clinical study of 142 CRC patients, Guo et al. found that ABCB5 mRNA transcripts are significantly enriched in patient peripheral blood specimens compared with those in non-CRC controls and correlate with CRC disease progression. Notably, ABCB5 regulates CRC invasiveness, at least in part by enhancing AXL receptor tyrosine kinase signaling [76]. Additionally, in glioblastoma and melanoma, high expression of drug efflux pumps, such as ABCG2 and ABCB5, is reported in CSCs [77,78].

### 4.2. Apoptosis

In addition to other mechanisms, CSC resistance to drug cytotoxicity is commonly associated with intrinsic or acquired defects and/or inefficient signaling in either the extrinsic or intrinsic pathway of apoptosis. Regarding the extrinsic pathway, lung, pancreatic, breast and glioma CSC models exhibit resistance to TRAIL-induced apoptosis by genetic and epigenetic silencing of pro-apoptotic factors, such as caspase 8 or c-FLIP, or by upregulation of TRAIL-R2 receptor, as reviewed by Fulda [79]. Regarding the intrinsic pathway, the role of Bcl-2 family members in tumorigenesis and cancer cell survival has been known for a long time, and its effects on CSC biology are well described. Bcl-2 is highly expressed in CD44^+^/CD24^-/low^ breast CSCs [80] and in quiescent leukemic CD34^+^ progenitor cells [81]. Similarly, in colon CSCs, high levels of Bcl-xL activity were found in a BH3 mimetic screen [82]. Reductions of Bcl-xL expression resulted in increased sensitivity to oxaliplatin and 5-fluorouracil (5-FU) [83]. Other members of the Bcl-2 family can be involved in CSC apoptotic escape, such as MCL-1, that was found to be downregulated by MiR-519d in cisplatin-resistance breast CSCs [84]. However, it is clear that the plethora of intrinsic and extrinsic apoptosis factors involved in the fine-tuning of the apoptotic process could be affected. Indeed, Rouhrazi et al., performing a quantitative real-time polymerase chain reaction (qRT-PCR) screen for 84 key apoptosis-related genes in zoledronic acid-resistant CD133^+^/CD44^+^ prostate CSCs, showed significant over/underexpression of a cluster of anti-apoptotic and pro-apoptotic genes [85]. Our group confirmed the increase in expression of antiapoptotic proteins in another preclinical model of zoledronic acid resistant prostate cancer cells in which the acquisition of CSCs features has been described. Interestingly, activation of the p38-MAPK in these cells was found to be a crucial mechanism in the regulation of several biological processes, such as antiapoptotic, prosurvival, proinflammatory and proangiogenic events, as well as EMT and invasion [86,87]. Similarly, a proteomic analysis of colonosphere cultures derived from resection specimens of liver metastases in patients with colon cancer highlighted that 20 of 32 proteins upregulated two-fold in CSCs were classified as regulating "Cell Death" [88]. From this perspective, in contrast to the observed CSC high apoptotic threshold, only combination therapies and drugs with pleiotropic effects could be effective at targeting the multiple resistance mechanisms. 

### 4.3. Alteration of DNA Damage Repair System

Since radiotherapy and the majority of chemotherapeutic agents induce DNA damage, perturbation of the DNA damage repair system is another well-described mechanism of CSCs to escape genotoxic effects. As reviewed by Vitale et al. various molecular alterations lead to a robust DNA damage response (DDR) in CSCs compared with the relatively more differentiated malignant cells in glioblastoma, breast, lung and CRC [73]. However, each CSC could establish a specific alteration that increases the basal dependence on specific DDR components for proliferation and survival. Moreover, each DDR protein is under the control of several factors and in turn modulates several phenomena such as ATM (ataxia-telangiectasia mutated). Indeed, Bao et al. highlight that the CSC population exhibits an upregulation of phosphorylated ATM, Chk2, RAD51 and RAD17, either at baseline or in response to radiation [89]. Moreover, Carruthers et al. indicated that CSCs possessed ATM-independent mechanisms for activation and maintenance of the G2/M checkpoint, whereas differentiated tumor cell populations appeared to be more reliant on ATM function for G2/M checkpoint integrity [90]. In addition, Zhang et al. showed that ZEB1 participated in an ATM-dependent mechanism in the DDR response in breast CSCs by stabilizing CHK1 [91].

### 4.4. Quiescence State

In addition to a robust DDR, CSCs display a persistent quiescence state. The notion that standard chemotherapeutic resistance results from the persistence of quiescent CSCs has emerged recently by genetic-fate mapping in several solid tumor types. A slow proliferative state is essential for the survival of cells (resistance) in the presence of oxaliplatin or temozolomide treatments in CRC and in glioblastoma, respectively [92,93]. Knowledge concerning activated or silenced mechanisms relying on this state could be useful to employ combinatorial therapeutic strategies to manipulate and to sensitize CSCs to chemotherapeutics. For example, activated TGF-β signaling (among others, responsible for triggering cytostatic signals) drives the dormancy of CSCs in mouse squamous cell carcinoma, leading to cisplatin resistance [94]. Similarly, a subpopulation of CSCs undergoing EMT is associated with a slow proliferative state that confers resistance to anti-proliferative drugs in a model of breast and skin cancer [95]. Moreover, Soeda et al. reported that inhibition of the p38 MAPK pathway led to an increase in EGFR expression but reduced proliferation and cell death induction and promoted maintenance of an undifferentiated state [96]. Recently, it has been demonstrated that leukemia stem cells express the highest levels of enhancer of zeste homolog 1 (EZH1) and 2 (EZH2), two histone-lysine N-methyltransferases that mediate methylation of histone H3 at lysine 27 (H3K27), to maintain the quiescent state. The key role of these two enzymes was confirmed, showing that inactivation of EZH1/2 eradicated quiescent leukemia stem cells, inducing cell cycle progression and differentiation [97]. Notably, EZH2 was also associated with radioresistance of glioma stem cells [98] as well as CD24^–/low^CD44^+^ population amplification in breast cancer upon treatment [99]. 

### 4.5. Metabolism Adaptation

In recent years, conventional wisdom indicates that CSC metabolism adaptation could play a main role in chemoresistance and radioresistance. Metabolism based on oxidative phosphorylation is crucial for the generation of energy needed to support the maintenance of tumors; however, this process also produces reactive oxygen species (ROS), which have the potential to cause stem cell dysfunction. For this reason, CSCs usually grow in a hypoxic niche and employ a glycolytic metabolism [100,101]. Additionally, CSCs are characterized by increased ROS levels, reduced oxidative damage and, thus, longer survival, potentially due to a combination of mechanisms that arise in the tumor, including the modulation of multiple antioxidative enzyme systems or redox-sensitive signaling pathways, such as NRF2, NF-κB, c-Jun, and HIFs, leading to increased expression of antioxidant molecules, as recently reviewed in detail by our group [102].

Another altered metabolic feature described in the CSC subpopulation is the high glycolytic metabolism recently demonstrated to be related to the high mitochondrial mass in MCF-7 breast cancer cells [103]. In detail, an unbiased proteomic approach has allowed the establishment that mitochondrial proteins are among the most strongly upregulated in cells overexpressing WNT1 and FGF3, which are responsible for the stemness phenotype. Interestingly, the mito-high MCF7 cells are also resistant to paclitaxel, resulting in little or no DNA damage [103]. Moreover, an enrichment in mitochondrial content has been associated with a higher DNA repair capacity in human breast cancer stem cells, suggesting that an increased mitochondrial mass may enable CSCs to cope efficiently with the action of certain anticancer drugs [104]. In line with these findings, a metabolic profile of CD34^+^ and CD34^−^ chronic myeloid leukemia (CML) cells, derived from fourindividuals by recording steady-state levels of 70 metabolites through liquid chromatography–mass spectrometry (LC–MS), highlighted a selective increase in glucose oxidation and anaplerosis, the process of replenishment of depleted metabolic pathway intermediates, in CML cells resistant to imatinib treatment [105].

### 4.6. Immune Evasion

Finally, a recently described CSC-resistance feature is immune evasion. CSCs have evolved sophisticated strategies to escape host immune surveillance that are targets of current therapeutic efforts (as reviewed by Fiori and Maccalli) [106,107]. A bioinformatic approach using The Cancer Genome Atlas (TCGA) revealed an enrichment of tumor-intrinsic WNT/β-catenin signaling in non-T cell inflamed tumors, providing a strong rationale for the development of pharmacologic inhibitors of this pathway with the aim of restoring immune cell infiltration and augmenting the immunotherapeutic response [108]. Molecularly, Agudo et al. showed that cycling epithelial stem cells, including Lgr5^+^ intestinal stem cells, as well as ovary and mammary stem cells, were eliminated by activated T cells, but quiescent stem cells in the hair follicle and muscle were resistant to T cell killing. Mechanistically, the authors highlighted that the quiescent stem cells downregulated the antigen presentation machinery, including MHC class I and TAP proteins, through the trans-activator NLRC5 [109]. Similarly, the slow-cycling CSC subpopulation in CRC, with the loss of the major histocompatibility complex by overexpression of costimulation molecules and CSC-specific antigens, were resistant to the cytotoxic effect of dendritic and cytokine-induced killer cells [110]. Interestingly, Peng et al. found that myeloid-derived suppressor cells (MDSC) promoted tumor formation by enhancing breast CSC-like properties as well as by suppressing T cell activation, claiming a cross-talk between MDSC and breast CSCs. Specifically, MDSC inducing IL6-dependent phosphorylation of STAT3 and activation of NOTCH through nitric oxide lead to prolonged STAT3 activation in CSCs [111]. However, our knowledge about specific immunological properties of distinct CSC populations is still limited and requires further study to implement new targetingtherapeutic strategies. 

Overall, these observations suggest that CSCs have several ways to escape anti-tumor approaches and that it is critical to identify CSC-specific alterations for targeting within a single tumor or rather to use drugs with pleiotropic effects to successfully target this difficult to kill subpopulation.

## 5. HDAC Inhibitors Are Able to Overcome Chemo-Resistance

As pleiotropic agents, HDACi can modulate a wide variety of molecules and target several related molecular pathways. Thus, it is obvious that this class of drug could affect many escape chemotoxicity strategies implemented by tumor cells and CSCs, which are described above. 

Indeed, it has been demonstrated that entinostat, as a type of HDACi, reverses cisplatin resistance, among various mechanisms, by the induction of apoptosis with an increase in cleaved PARP and a decrease in MDR1 in esophageal squamous cell carcinoma [112]. Similarly, Zhao et al. observed that specific inhibition of HDAC8 mediates the upregulation of miR-137 and inhibition of MDR1 to sensitize neuroblastoma cells to doxorubicin (Dox) [113]. In a lung cancer cell model, To et al. demonstrated that belinostat reverts cisplatin resistance by the inhibition of both ABCC2 and the DNA repair gene ERCC1 [114].

Concerning P-glycoprotein (P-gp), another crucial drug efflux transporter, although it has been reported that HDACi upregulates P-gp in colorectal cancer cells through STAT3 induction and ABCB1 posttranscriptional stabilization [115], Tomono et al. demonstrated that HDACi inhibited Snail-induced activation of P-gp in lung cancer Snail-overexpressing cells [116]. Moreover, in squamous carcinoma cell lines, Chikamatsu et al. reported that the expression of CSC markers, such as CD44 and ABCG2, decreased upon vorinostat and TSA treatment and that the combination of these HDACi with cisplatin or docetaxel had a synergistic cytotoxic effect [117]. 

As pointed out above, HDACi usually work as sensitizers and modulators of the entire gene pattern, synergizing with several chemotherapeutics and molecular targeted agents. Thus, the doses used in *in vitro* and *in vivo* experiments are not expected to provide clear apoptosis induction. Nevertheless, Aztopal et al. reported that relatively low doses (2.5 and 5 mM) of VPA prevented mammosphere formation, inducing apoptosis [118]. Similarly, Di Pompo et al. reported that new compounds with selective HDACi activity affect CSCs generated from three different histotypes of human sarcomas, inducing, among others, apoptosis [119]. The author demonstrated that concurrent class I and IIb HDAC inhibition is crucial to obtain anticancer effects. In pancreatic CSCs, vorinostat epigenetically restores the expression of miR-34a, leading to apoptosis through caspase-3/7 activation [120]. Moreover, HDAC1 inhibition contributes to NANOG-mediated TRIM17 and *NOXA* gene expression, leading to a downregulation of antiapoptotic MCL-1, conferring immuno- and chemo-sensitization [121].

Since a clear characterization of CSCs has not yet been defined completely, we believe that the majority of findings regarding overcoming chemoresistance were not described in a specific cell subpopulation; therefore, we have also reviewed the literature on HDACi and their ability to sensitize resistance in subpopulations in many cancer types regardless of stem-like features.

Fluoropyrimidine-based therapy still represents a classic therapeutic strategy in several solid tumors, such as colorectal, breast and pancreatic cancers; however, chemo-resistance remains a big open question to resolve. Resistance to 5-FU or to its pro-drugs may result from deficient drug uptake, alterations of targets, activation of DNA repair pathways, resistance to apoptosis andalterations of the tumor microenvironment. 

We and others have previously demonstrated synergistic antitumor effects of different HDACi in combination with fluoropyrimidines in different tumors, such as breast, colorectal [48,122,123,124,125] and head and neck squamous cell carcinomas (HNSCC) [126]. Our results demonstrated that the synergistic interaction between HDACi and 5-FU is dependent on both the downregulation of thymidylate synthase (TS), the key enzyme in the mechanism of action of 5-FU, and on the upregulation of thymidine phosphorylase (TP), the key enzyme converting capecitabine to 5-FU. Interestingly, HDAC3 is the HDAC isoform principally involved in TP upregulation. These observations could be clinically relevant since HDAC3 hasrecently emerged as a critical anticancer target [127,128,129], and more selective HDAC3 inhibitors may have a more favorable side-effect profile compared with class-I or non-selective HDACi. Intrinsic or acquired resistance to 5-FU is often related to TS protein overexpression. Indeed, high levels of TS expression have been correlated with poorer overall patient survival in several tumors [130]. Interestingly, we have previously demonstrated a synergistic antitumor effect of vorinostat with 5-FU in CRC cells selected for resistance to 5-FU (HT29FU cells) and in cells carrying an amplification of the TS gene (H630-R10 cells), suggesting a potential mechanism by which vorinostat may overcome the resistance to 5-FU [122]. Similarly, we recently showed that VPA/capecitabine combination treatment synergizes with radiotherapy (RT), confirming the modulation of both TS and TP protein levels by VPA in CRC models, even in the presence of RT [48]. Based on these data, a phase 1/2 study is currently ongoing exploring VPA at an antiepileptic dosage, in combination with capecitabine, during preoperative radiotherapy in locally advanced rectal cancers patients (V-shoRT-R3 trial) [131]. Notably, data from the completed phase 1 of the study support the feasibility of VPA in combination with chemoradiotherapy [132].

In the same context, Huang et al. demonstrated that TSA was able to decrease colon CSC properties and, in combination with 5-FU, suppress colon cancer viability *in vitro* and colon tumorigenesis *in vivo* [133].

Another challenging class of chemotherapeutics is represented by the “platinum complexes”, which includes anticancer drugs such as oxaliplatin, cisplatin and carboplatin. Cisplatin is currently employed for several tumors, such as testicular, ovarian, bladder, head and neck, esophageal, small and non-small-cell lung, cervical, stomach and others, regiments containing oxaliplatin, with or without a biologic agents in combination, are the optimal choice for metastatic CRC treatment, leading to sufficient disease reduction and allowing patients to become eligible for resection of metastatic diseases.

However, cisplatin or oxaliplatin treatments are associated with drug resistance and several adverse side effects. For these reasons, a combinational strategy aimed to revert resistance, improve outcomes and reduce side effects, could be of great benefit. Unpublished results from our group demonstrated that VPA induces cellular differentiation and sensitization of colorectal CSCs to oxaliplatin. In details, by using CRC primary spheroid cultures, transduced with a TOP-GFP Wnt reporter, we monitored apoptosis and cell proliferation in both differentiated cells and CSCs within the same population, treated with VPA alone and/or standard chemotherapy [50]. Based on these preliminary results, a randomized phase II trial has been designed by our group to evaluate whether the combination of VPA with bevacizumab and oxaliplatin/fluoropyrimidine regimens (mFOLFOX6/mOXXEL) prolongs progression-free survival (PFS) compared with bevacizumab and oxaliplatin/fluoropyrimidine regimens alone, as the first-line treatment in patients with metastatic CRC with mutation of RAS (Revolution Trial–Randomized phase II study of VPA in combination with bevacizumab and Oxaliplatin/fLUoropyrimidine regimens in patients with ras-mutated metastaTIc cOlorectal cancer). Correlative studies will be performed on patient materials to study the impact of the treatment on the CSCs population.

Cisplatin resistance has been reported in HNSCC cells to be related to enhanced stem cell properties, tumor metastasis, and increased HDACs expression [134]. Wang et al. demonstrated that cisplatin treatment, but not paclitaxel and doxorubicin treatment, result in the enrichment of CSCs, conferring multidrug resistance in NSCLC cell lines by the induction of TRIB1 and HDACs [135]. It has been demonstrated that TRIB1 enhances histone deacetylase 1 (HDAC1)-mediated p53 deacetylation and decreases DNA binding of p53, decreasing its tumor suppressor activity [136]. Moreover, high levels of TRIB1 show a significantly poorer prognosis in CRC patients, in NSCLC cisplatin-treated patients and in a Chinese Han population with pancreatic cancer [135,137,138]. In metastatic NSCLC, pancreatic and bladder cancer cisplatin treatment is associated with gemcitabine. In this regard, it has been described that the pan-HDACi CG200745, decreasing the transcript for multidrug resistance protein (MRP) 4, a member of the MRP/ABCC subfamily of the ATP-binding cassette, controls drug efflux and sensitivity in gemcitabine-resistant pancreatic cancer cells [139], for which gemcitabine-based regimens are still the major treatment. Moreover, the inhibition of HDACs 1, 7 and 8 by TSA or vorinostat results in an upregulation of e-cadherin in PDAC cells and downregulation of Oct-4, Sox-2, and Nanog, as well as inhibition of PDAC tumor sphere formation, resulting in a strong potentiation of gemcitabine therapeutic activity [16]. Interestingly, overexpression of HDAC7 has been demonstrated in pancreatic cancer and suggested as clinical biomarker for pancreatic cancer diagnosis and prognosis [140].

Additionally, CG200745, by inducing miR-509-3p expression, selectively targets Hippo signaling in cholangiocarcinoma cells and synergistically interacts with conventional chemotherapeutic drugs, including gemcitabine, cisplatin, oxaliplatin and 5-FU, even enhancing the sensitivity of gemcitabine-resistant cholangiocarcinoma cells to these drugs [141]. However, although co-treatment of biliary tract cancer cells with HDACi, including TSA, VPA or vorinostat, and gemcitabine suppresses EMT with tolerable cytotoxicity [62,142], it has also been reported that HDACi increase the expression of both e-cadherin and vimentin in different cholangiocarcinoma cell lines, suggesting that further analyses are needed before using these drugs in the clinic for the treatment of this tumor [142]. The preclinical synergistic interaction between HDACi and gemcitabine has also been described in leiomyosarcoma, for which Lopez et al. demonstrated that the selective class I/IV HDACi mocetinostat combined with gemcitabine exhibits synergistic effects *in vitro* and *in vivo* [143]. 

Interestingly, in a phase I study in which vorinostat was combined with carboplatin and gemcitabine in women with recurrent, platinum-sensitive epithelial ovarian, fallopian tube, or peritoneal cancer, despite no maximum tolerated dose determined due to toxicities, six of the seven patients evaluable for RECIST (Response Evaluation Criteria in Solid Tumours) assessment had partial responses (PR) [144]. These results indicate that the use of other HDACi with a better safety profile, such as VPA, could be further investigated in combination with gemcitabine and/or platinum compounds.

It has been already described in the literature that HDACi modulate the EGFR family in several ways, as also reported by our group [145,146]. This evidence explains how HDACi improve the EGFR-based target therapy and how they overcome the drugresistance that is commonly reported after the first anti-EGFR target therapy cycles of treatment. Indeed, Wang et al. showed that HDAC6 overexpression confers resistance to gefitinib via the stabilization of EGFR. Moreover, inhibition of HDAC6 by CAY10603, a selective inhibitor, represses the proliferation and synergizes with gefitinib to induce apoptosis in lung adenocarcinoma cell lines, via the destabilization of EGFR [147]. In the same model of NSCLC, it has been reported that the knockdown of HDAC1 sensitize resistant cells to paclitaxel *in vitro* and that SNOH-3, a selective HDAC1 inhibitor, induces apoptosis and suppresses angiogenesis in preclinical models [148]. In addition, we found that vorinostat or VPA sensitize primary NSCLC cell lines to anti-ErbB3 monoclonal antibody by modulating the EGFR family also in 2D and in 3D culture models, enriched in CSCs [47]. Interestingly, a phase I study of CUDC-101, a multitarget inhibitor of HDACs, EGFR, and HER2, in combination with chemoradiation in patients with HNSCC, showed an increase of 1.5 years in median duration of response and 9/15 patients free with increased PFS [149]. Other examples of combined treatment of HDACi with targeted drugs have also been reported. For example, Gruber et al. showed that 4SC-202, a class I HDACi, abrogates GLI activation and Hh target gene expression in both SMO-inhibitor-sensitive and -resistant cells. Significantly, it has been reported that treatment with SMO inhibitors leads to rapid and frequent development of drug resistance in basal cell carcinoma and medulloblastoma [68]. 

Finally, it is important to highlight that, despite a good and durable clinical benefit obtained by immune checkpoint blockers (ICB) in several tumors, particularly “hot” or “immunogenic tumors”, in other solid tumors, the responses with ICB are quite modest, representing a critical therapeutic challenge [150]. A possible solution to address such challenges could be to combine ICB with specific drugs aimed to prime the immune response and increase the tumor immune profile. Published data suggest that HDACi enhance the immunogenicity of cancer cells. Indeed, HDACi are involved in the regulation of NK cell-activating ligands, MHC class I and II molecules, elevation of NK and CD8+ cytotoxicity and proinflammatory cytokines, and modulation of Treg and Treg Foxp3 expression [151]. Terranova-Barberio et al. showed that HDACi treatment leads to a significant decrease in tumor growth through epigenetic priming of the immune system, with increased tumor antigen presentation and immune cell activation. Interestingly, both pan-HDACi and class-selective HDACi promote an upregulation of PD-L1 and HLA-DR in triple negative breast cancer cell models when co-cultured with peripheral blood mononuclear cells, associated with a downregulation of CD4^+^ Foxp3^+^ Treg *in vitro* and *in vivo* [152]. In line with this finding, Miyashita et al. found that the combination of low-dose VPA and gemcitabine enhances the susceptibility of the PANC-1 cell line to γδT cell-mediated tumor cell lysis through the upregulation of major histocompatibility complex class 1-related chain molecules [153]. Interestingly, several phase I and II clinical trials, based on HDACi treatment in combination with immunotherapy, are recruiting with the aim to identify optimal biological dosing strategies [151]. The first results are not yet available, and additional preclinical studies need to be performed to disclose the mechanisms underlying the controversial HDACi-dependent effects.

## 6. Conclusions and Future Perspectives

In solid cancers, multiple factors contribute to the failure of commonly used therapies, leading to relapse. Here, we emphasized the aspects related to CSCs and their involvement in this phenomenon, reviewing what is known in the literature. By highlighting the pathways and mechanisms responsible for the resistance to the commonly used chemotherapeutics, we suggest that only the addition of pleiotropic molecules will target the CSCs population efficiently. From this perspective, HDACi could be among the best candidate drugs. We have reviewed the role of HDACi in solid cancers, specifically in the CSC subpopulation, and have pointed out some mechanisms by which HDACi are able to overcome drug resistance. In clinicaltrials.gov, 377 registered studies in solid tumors with HDACi were retrieved. Most of them are phase-I and phase-II trials and several are currently recruiting as a single agent or in combination therapies (Table 2). Only seven phase-III studies are reported and only one have published results (Table 3). In the latter study, the vorinostat effect was evaluated in monotherapy, in a double-blind, randomized placebo-controlled trial, involving 90 international centers and enrolling 661 patients with measurable advanced malignant pleural mesothelioma and disease progression after one or two previous systemic regimens. The study was negative, with no statistical significant difference (median overall survival of 30.7 weeks; 95% CI 26.7–36.1) versus the placebo arm (27.1 weeks; 95% 23.1–31.9) [154] confirming negative results obtained by this class of agent in monotherapy in several solid tumor types [155]. Another phase -III trial (ClinicalTrial.gov identifier: NCT00473889) exploring the combination of vorinostat plus carboplatin and paclitaxel in NSCLC patients was terminated due to negative results and increased toxicity, although a phase II trial demonstrated that vorinostat was able to improve the efficacy of chemotherapy [156]. Even as single agent, drug-induced side-effects of HDACi were observed, associated with several toxicities including cardiotoxicities, hematological and gastrointestinal toxicities [157]. However, despite being able to affect a multitude of physiological cellular process, thus being potentially very toxic, several HDACi have gained FDA approval for use in hematological malignancies [155].

Moreover, we strongly believe that HDACi should be use in clinical practice as biological modifiers and not as cytotoxic drugs. Consequently, by contrast with clinical trials conducted so far, low doses of these drugs should be used to avoid direct cytotoxic effects. In addition, we believe that the therapeutic window of this class of drug is only in combination with standard chemotherapeutics. 

On these bases, further clinical and preclinical investigations should be conducted to better disclose the mechanisms by which HDACi modulate several signaling pathways in different tumors. In summary, as highlighted in this review, the promising data obtained until now could represent the foundation to test novel combinatorial therapeutic strategies, where HDACi would be combined with commonly used drugs to improve therapeutic efficacy in solid cancer tumors.

## Figures and Tables

**Table 1:** Prevalence of CSCs-activated signaling pathways in solid tumors accordingly with outcome: Results obtained by R2 on line tool. Clustering the patients in dead and alive for each solid cancer, the values are indicative of how enriched in dead patients are the Hippo, Wnt, Jak/STAT, TGFb, Notch, Hedgehog pathways, based on gene expression data. The values are between 0 and 1 where 0 is the best over-representation. The color scale (from violet to white) groups the 6 CSCs pathways from the highest to the lowest enriched, among each TCGA (The Cancer Genome Atlas) dataset.

			Patient Status		CSCs-Activated Signaling Pathways					
Solid Cancer	TCGA_Id	Whole Sample	Alive	Dead	Hippo	Wnt	Jak/STAT	TGF	Notch	Hedgehog
Mixed Colon Adenocarcinoma	COAD	174	140	15	0.880	0.310	0.470	0.640	0.950	0.910
Rectum adenocarcinoma	READ	95	87	7	0.360	0.800	0.00041	0.150	1.000	0.360
Pancreatic adenocarcinoma	PAAD	178	119	59	0.270	0.330	0.430	0.550	0.390	0.520
Lung Adenocarcinoma	LUAD	515	389	126	0.900	0.300	0.400	0.210	0.800	0.420
Lung Squamous Cell Carcinoma	LUSC	81	61	19	0.390	0.530	0.610	0.440	0.580	0.620
Prostate Adenocarcinoma	PRAD	497	489	8	0.016	0.300	0.670	0.210	0.360	0.360
Stomach adenocarcinoma	STAD	415	336	79	0.260	0.410	0.500	0.310	0.460	0.520
Liver Hepatocellular Carcinoma	LIHC	371	282	89	0.840	0.510	0.580	0.680	0.550	0.680
Kidney Renal Clear Cell Carcinoma	KIRC	533	363	160	0.780	0.620	0.810	0.120	0.080	0.950
Head Neck Squamous Cell Carcinoma	HNSC	520	353	167	0.140	0.006	0.030	0.930	0.440	0.500
Cervical Squamous Cell Carcinoma	CESC	305	244	60	0.400	0.610	0.520	0.630	0.480	0.530
Bladder Urothelial Carcinoma	BLCA	408	300	108	0.260	0.100	0.500	0.310	0.440	0.520
Sarcoma	SARC	259	184	75	0.580	0.680	0.740	0.620	0.720	0.750
Breast Invasive Carcinoma	BRCA	1097	992	104	0.100	0.300	0.400	0.670	0.800	0.000008
Glioblastoma	TARGET_NBL	153	52	99	0.840	0.400	0.002	0.410	0.550	0.160
Skin Cutaneous Melanoma	SKCM	470	313	156	0.360	0.500	0.580	0.410	0.550	0.600

**Table 2:** Phase and status of HDACi clinical trials: clinical trials registered in https://clinicaltrials.gov/ in solid tumors with HDACi.

	Recruiting	Active, Not Recruiting	Not Yet Recruiting	Completed	Terminated	Suspended	Withdrawn	Unknown Status	Sum
Early Phase 1	2	0	0	0	0	0	1	1	4
Phase 1	24	34	8	109	45	2	5	3	230
Phase 2	15	16	3	55	37	0	1	3	130
Phase 3	1	2	0	1	1	0	0	2	7
Not Applicable	0	1	0	4	0	0	1	0	6

**Table 3:** Phase-III HDACi clinical trials: characteristics of Seven phase-III clinical trials registered in https://clinicaltrials.gov/ that involve HDACi.

Phase	Title	Status	Completion Date	Description	Condition	Url
Phase 3	Hydralazine Valproate for Ovarian Cancer	Unknown status	December 2009	Randomized, double-blind phase III trial. A total of 211 patients (alpha 0.5, power 0.8) with cisplatin-resistant recurrent or persistent cancer will be randomized to topotecan + placebo or topotecan + hydralazine + valproate for 6 courses every 4 weeks.	Ovarian Cancer	https://ClinicalTrials.gov/show/NCT00533299
Phase 3	Hydralazine Valproate for Cervical Cancer	Unknown status	December 2010	Randomized, double-blind phase III trial. A total of 143 patients (alpha 0.5, power 0.8) with metastatic, persistent or recurrent cervical cancer without previous systemic treatment will be randomized to cisplatin topotecan + placebo or cisplatin topotecan hydralazine valproate for 6 courses every 3 weeks.	Metastatic Cervical Cancer	https://ClinicalTrials.gov/show/NCT00532818
Phase 3	Anticancer Activity of Nicotinamide on Lung Cancer	Active, not recruiting	June 2020	Randomized Double-blinded Comparative Trial to study the Add-on Activity of Combination Treatment of Nicotinamide on Progression Free Survival for EGFR Mutated Lung Cancer Terminal Stage Patients Being Treated With Gefitinib or Erlotinib.	Non-Small-Cell Lung Carcinoma	https://ClinicalTrials.gov/show/NCT02416739
Phase 3	Exemestane With or Without Entinostat in Treating Patients With Recurrent Hormone Receptor-Positive Breast Cancer That is Locally Advanced or Metastatic	Active, not recruiting	-	Randomized phase III trial studies exemestane and entinostat to see how well they work compared to exemestane alone in treating patients with hormone receptor-positive breast cancer that has spread to nearby tissue or lymph nodes or another place in the body.	Breast Adenocarcinoma	https://ClinicalTrials.gov/show/NCT02115282
Phase 3	Exemestane With or Without Entinostat in Chinese Patients With Hormone Receptor-Positive, Locally Advanced or Metastatic Breast Cancer	Recruiting	August 2021	A Randomized Phase III Clinical Study of Entinostat/Placebo in Combination With Exemestane in Chinese Patients With Hormone Receptor-positive Advanced Breast Cancer.	Advanced Breast Carcinoma	https://ClinicalTrials.gov/show/NCT03538171
Phase 3	A Clinical Trial of Vorinostat (MK0683, SAHA) in Combination With FDA Approved Cancer Drugs in Patients With Advanced Non-Small Cell Lung Cancer (NSCLC)(0683-056)	Terminated	December 2008	A Phase II/III Randomized, Double-Blind Study of Paclitaxel Plus Carboplatin in Combination With Vorinostat or Placebo in Patients With Stage IIIB (With Pleural Effusion) or Stage IV Non-Small-Cell Lung Cancer (NSCLC).	Stage IIIB or IV Non-Small Cell Lung Cancer	https://ClinicalTrials.gov/show/NCT00473889
Phase 3	Suberoylanilide Hydroxamic Acid (Vorinostat, MK-0683) Versus Placebo in Advanced Malignant Pleural Mesothelioma (0683-014 AM5, EXT1)	Completed	November 2011	A Phase III, Randomized, Double-Blind, Placebo-Controlled Trial of Oral Suberoylanilide Hydroxamic Acid (Vorinostat, MK-0683) in Patients With Advanced Malignant Pleural Mesothelioma Previously Treated With Systemic Chemotherapy.	Mesothelioma/Lung Cancer	https://ClinicalTrials.gov/show/NCT00128102
